# Identification of a novel merozoite surface antigen of *Plasmodium vivax*, PvMSA180

**DOI:** 10.1186/s12936-017-1760-9

**Published:** 2017-03-28

**Authors:** Fauzi Muh, Jin-Hee Han, Myat Htut Nyunt, Seong-Kyun Lee, Hye-Yoon Jeon, Kwon-Soo Ha, Won Sun Park, Seok-Ho Hong, Md Atique Ahmed, Sunghun Na, Eizo Takashima, Takafumi Tsuboi, Eun-Taek Han

**Affiliations:** 1 Department of Medical Environmental Biology and Tropical Medicine, School of Medicine, Kangwon National University, Chuncheon, Gangwon-do Republic of Korea; 2 Department of Medical Research, Yangon, Republic of the Union of Myanmar; 3 Department of Cellular and Molecular Biology, School of Medicine, Kangwon National University, Chuncheon, Gangwon-do Republic of Korea; 4 Department of Physiology, School of Medicine, Kangwon National University, Chuncheon, Gangwon-do Republic of Korea; 5 Department of Internal Medicine, School of Medicine, Kangwon National University, Chuncheon, Gangwon-do Republic of Korea; 6 Department of Obstetrics and Gynecology, School of Medicine, Kangwon National University, Chuncheon, Gangwon-do Republic of Korea; 7 Division of Malaria Research, Proteo-Science Center, Ehime University, Matsuyama, Ehime 790-8577 Japan

**Keywords:** *Plasmodium vivax*, MSA180, Humoral immune response, Merozoite surface protein, Haplotype

## Abstract

**Background:**

Although a number of *Plasmodium vivax* proteins have been identified, few have been investigated as potential vaccine candidates. This study characterized the *Plasmodium vivax* merozoite surface antigen 180 (PvMSA180, PVX_094920), a novel *P*. *vivax* antigenic protein.

**Methods:**

The target gene was amplified as four overlapping domains (D1, D2, D3 and D4) to enable expression of the recombinant protein using cell-free and bacterial expression systems. The recombinant PvMSA180 proteins were used in protein microarrays to evaluate the humoral immune response of 72 vivax-infected patients and 24 vivax-naïve individuals. Antibodies produced in mice against the PvMSA180-D1 and -D4 domains were used to assess the subcellular localization of schizont-stage parasites with immunofluorescence assays. A total of 51 *pvmsa180* sequences from 12 countries (41 sequences from PlasmoDB and 6 generated in this study) were used to determine the genetic diversity and genealogical relationships with DNAsp and NETWORK software packages, respectively.

**Results:**

PvMSA180 consists of 1603 amino acids with a predicted molecular mass of 182 kDa, and has a signal peptide at the amino-terminus. A total of 70.8% of patients (51/72) showed a specific antibody response to at least one of the PvMSA180 domains, and 20.8% (15/72) exhibited a robust antibody response to at least three of the domains. These findings suggest that PvMSA180 is targeted by the humoral immune response during natural infection with *P*. *vivax*. Immunofluorescence analysis demonstrated that PvMSA180 is localized on the merozoite surface of schizont-stage parasites, and *pvmsa180* sequences originating from various geographic regions worldwide showed low genetic diversity. Twenty-two haplotypes were found, and haplotype 6 (Hap_6, 77%) of *pvmsa180* was detected in isolates from six countries.

**Conclusions:**

A novel *P*. *vivax* surface protein, PvMSA180, was characterized in this study. Most of *P*. *vivax*-infected patients had specific antibodies against particular antigenic domains, indicating that this protein is immunogenic in naturally exposed populations. Genetic analysis of worldwide isolates showed that *pvmsa180* is less polymorphic than other well-known candidates and that some haplotypes are common to several countries. However, additional studies with a larger sample size are necessary to evaluate the antibody responses in geographically separated populations, and to identify the function of PvMSA180 during parasite invasion.

**Electronic supplementary material:**

The online version of this article (doi:10.1186/s12936-017-1760-9) contains supplementary material, which is available to authorized users.

## Background

Malaria is a public health problem in countries in which it is endemic [1]. In 2015, the number of cases and deaths was 214 million and 438,000, respectively [2]. *Plasmodium vivax* causes 50% of all malaria cases globally [3], and is prevalent in the tropics and subtropics [4]. A malaria vaccine shows promise for controlling malaria [5]; however, the antigenic diversity and immune-evasion ability of *Plasmodium* has hampered vaccine development [6]. Molecules expressed on the merozoite surface, such as apical membrane antigen-1 (AMA1), merozoite surface protein-1 (MSP1), and Duffy binding protein, have been the focus of vaccine development efforts [7].

Bioinformatic and genome analysis of *P*. *vivax* have led to the identification of malaria antigens, few of which have been investigated as vaccine candidates [8–10]. MSPs, such as MSP-1, MSP-9, MSP-4 and MSP-5, have been identified as vaccine candidates [11]. Some hypothetical proteins have been identified as vaccine candidates based on coiled coil structure [10]. Moreover, several proteins of *P. vivax* that are expressed on the surface or in apical organelles, including MSPs, rhoptry-associated membrane antigen, glycosylphosphatidylinositol (GPI)-anchored micronemal antigen and AMA1, have been proposed as vaccine candidates due to their involvement in merozoite invasion or the longevity of the antibody response [12–16]. Due to the limitations of *P*. *vivax* in vitro culture systems, fewer surface proteins have been identified in this pathogen than in *P*. *falciparum*. In fact, a number of *P*. *vivax* surface proteins have been identified based on their orthologues in *P*. *falciparum* [9, 10, 15, 17], and the antibody responses to them have been investigated [18–20].

One of hypothetical proteins, named *P*. *vivax* merozoite surface antigen 180 (PvMSA180) was previously identified [21]. Of the 96 *P*. *vivax* blood-stage proteins, 18 (including PvMSA180) elicited robust antibody responses [21]. Thus, this study has characterized PvMSA180, which is immunogenic in naturally exposed populations, and determined its subcellular localization in *P*. *vivax*.

## Methods

### Sample and serum information

Blood samples were collected from 72 patients in 2012 in the Shwe Kyin area of Myanmar (mean age, 24.5 years; range, 18–42 years). The patients were confirmed to have *P*. *vivax* malaria using the malaria rapid diagnostic test (SDFK80; Standard Diagnostics, Gyeonggi, Korea) and microscopy. Samples were centrifuged and the serum was separated. Serum samples from 24 healthy malaria-naïve individuals residing in non-endemic areas in the Republic of Korea (ROK) were also collected and used as controls.

### Amplification of full-length *pvmsa180*

Parasite genomic DNA was extracted using a Qiagen DNA Extraction Kit (Hilden, Germany). The *pvmsa180* (PVX_094092) sequence was obtained from PlasmoDB (http://plasmodb.org/). Full-length *pvmsa180* was amplified from five Myanmar and one South Korean isolate using the forward primer 5′-GATGACGACACAAACAAAAGGG-3′ and reverse primer 3′-CGCGGCGTAGTTGATGTG-5′. Full-length *pvmsa180* was amplified by PCR using high-fidelity *Thermococcus kodakaraensis* (KOD) DNA polymerase (Toyobo, Osaka, Japan) under the following conditions: 2.0 μL DNA template, 0.4 U KOD DNA polymerase, 0.25 mM of each primer and 500 μM of each dNTP, in a final volume of 20 mL. The cycling conditions were 94 °C for 2 min, followed by 35 cycles at 94 °C for 15 s, at 58 °C for 30 s, at 68 °C for 4.5 min, and a final extension at 68 °C for 10 min.

### Recombinant PvMSA180 expression

PvMSA180 was divided into four fragments and expressed using a cell-free system. The four fragments of *pvmsa180* were amplified under the aforementioned conditions, with the exception of a final extension for 1.5 min, using the following In-fusion primers:

D1-F: 5′-GGGCGGATAT ***CTCGAG***GATGACGACACAAACAAAAGGG-3′ and D1-R: 5′-GCGGTACCCGG ***GATCC***TTACCCGACATAGTACATTTGCTCA-3′; D2-F: 5′-GGGCGGATAT ***CTCGAG***AAGCTGCACCCAAAGAAGC-3′ and D2-R: 5′-GCGGTACCCGG ***GATCC***TTACATCTTCTCGTACAACAGCATATCA-3′; D3-F: 5′- GGGCGGATAT ***CTCGAG***GTGCTGGAGCTAGTAAATAATGATATG-3′ and D3-R: 5′- GCGGTACCCGG ***GATCC***TCAAAAGGCGCACTTCAAACTCA-3′; D4-F: 5′-GGGCGGATAT ***CTCGAG***GTGCGAAAAGAAAATGGAC-3′ and D4-R: 5′-GCGGTACCCGG ***GATCC***TCACGCGGCGTAGTTGATGTG-3′. Underlining indicates the 15 base pair (bp) homologous vector site; italicized, bolded, and underlined text indicates the *Xho*I and *BamH*I sites. The PCR products were cloned into the pEU-E01-His-Tev-N2 vector (Cell-Free Sciences, Matsuyama, Japan) using the In-fusion cloning kit (Clontech, Palo, Alto, CA, USA), and expressed using a wheat germ cell-free system (Cell-Free Sciences) [15, 22–24]. The crude recombinant proteins were used for immunoscreening of serum samples. Recombinant PvMSA180-D1 and PvMSA180-D4 proteins were expressed in *Escherichia coli*. PvMSA180–D1 was expressed using the pGEX-4T-2 expression vector (GE Healthcare, Upsala, Sweden) with a glutathione S transferase (GST) tag, and PvMSA180-D4 was expressed using the pET-28a (+) expression vector (Invitrogen, Carlsbad, CA, USA) with a His-tag. The target domains were amplified using In-fusion primers and High-Fidelity DNA Polymerase (Toyobo), and cloned into the appropriate expression vectors. Positive clones confirmed by DNA sequencing analysis were transformed into *E*. *coli* BL21(DE3) cells (Invitrogen). When the cultures reached an optical density of 0.6, expression of the recombinant D1 and D4 fragments was induced by addition of 0.1 and 0.3 mM isopropyl-β-d-thiogalactopyranoside, respectively. The GST-tagged proteins were purified using glutathione Sepharose 4B (GE Healthcare) and 6 His-tagged proteins using nickel-nitrilotriacetic acid (Ni–NTA) (Qiagen), according to the manufacturer’s instructions. The purity of the recombinant proteins was evaluated by sodium dodecyl sulphate–polyacrylamide gel electrophoresis (SDS-PAGE) and Western blotting.

### SDS-PAGE and Western blotting

The recombinant PvMSA180 proteins were resolved on 13% SDS-PAGE gels under reducing conditions, and then electrotransferred to 0.45 µm PVDF membranes (Millipore, Billerica, MA, USA) in semi-dry transfer buffer (50 mM Tris, 190 mM glycine, 3.5 mM SDS, 20% methanol) at a constant current of 360 mA for 40 min using a semi-dry blotting system (ATTO Corp., Tokyo, Japan). Recombinant PvMSA180-D1 and PvMSA180-D4 (1 µg each) and PvDBP-RII (0.5 µg) were used to assay antibody responses. The membranes were blocked in 5% skim milk and then incubated with a primary anti-GST antibody (1:10,000), anti-pentahistidine antibody (1:2000), mouse immune serum, or pooled patient serum (1:100), followed by incubation with a secondary IRDye^®^ goat anti-mouse (1:10,000 dilution) or IRDye^®^ goat anti-human (1:20,000) (LI-COR^®^ Bioscience, Lincoln, NE, USA) antibody. An Odyssey infrared imaging system and the accompanying software (LI-COR^®^ Bioscience) were used for data analysis.

### Protein microarray

Slides were coated with amine solution [21] and used to screen the serum of 72 vivax-infected patients and 24 healthy individuals by protein microarray. The crude recombinant proteins expressed using the wheat germ cell-free system were applied in duplicate and incubated for 2 h at 37 °C, followed by blocking in 5% BSA in phosphate-buffered saline (PBS) with 0.1% Tween 20 (PBS-T) and incubation for 1 h at 37 °C. Diluted serum (1:25) was added to a slide harbouring recombinant proteins and incubated for 1 h, followed by addition of an Alexa 647-conjugated goat-anti-human IgG (10 µg/mL, Invitrogen). Then slides were imaged using a fluorescence scanner (ScanArray Express, PerkinElmer, Boston, MA, USA). The cut-off value was equal to the mean fluorescence intensity (MFI) plus two standard deviations (SDs) of the negative samples. Normalized MFI values were calculated from the MFI/cut-off values.

### Production of mice antibody

Female BALB/c mice at 6–8 weeks of age (DBL, Seoul, ROK) were injected intraperitoneally with 30 µg recombinant PvMSA180-D1 and -D4 in PBS with complete Freund’s adjuvant (Sigma-Aldrich, St. Louis, MO) in a final volume of 100 µL, and subsequently boosted with incomplete Freund’s adjuvant. Three mice were injected three times with each of the D1 and D4 fragments of PvMSA180 at 2-week intervals. Sera were collected 2 weeks after the final boost. All the animal experimental protocols were approved by the Institutional Ethics Committee and were conducted in accordance with the Ethical Guidelines for Animal Experiments of Kangwon National University (ROK).

### Immunofluorescence assays

*Plasmodium vivax* parasites were collected from patients with malaria in Thailand and spotted onto eight-well glass slides. The slides were fixed in ice-cold acetone for 10 min, dried, and blocked in 5% BSA in PBS-T at 37 °C for 30 min. Then the slides were incubated (dual-labelled) with rabbit anti-PvMSP1-19 (1:50 dilution), rabbit anti-PvDBP (1:50 dilution), rabbit anti-PvRAMA (1:50 dilution), and mouse anti-PvMSA180 (1:100) as primary antibodies at 37 °C for 1 h. Next, the slides were stained with Alexa Fluor 568-conjugated anti-rabbit IgG or Alexa Fluor 488-conjugated goat anti-mouse IgG as secondary antibodies (Invitrogen), and nuclei were stained with 4′,6-diamidino-2-phenylindole (DAPI, Invitrogen) at 37 °C for 30 min. The slides were mounted in ProLong Gold antifade reagent (Invitrogen) and visualized under oil immersion using a confocal laser scanning microscope (FV200Olympus, Tokyo, Japan) equipped with 20× dry and 60× oil objectives. Images were captured using the FV10-ASW 3.0 viewer software.

### Sequence diversity and haplotype analysis of *pvmsa180* in isolates obtained worldwide

The *pvmsa180* sequences obtained in this study were compared to those of 46 isolated worldwide in the PlasmoDB database. *Pvmsa180* sequences from 12 countries (South Korea, Myanmar, Salvador, Peru, Colombia, Mexico, Thailand, Brazil, India, Papua New Guinea, North Korea, and China) were included in the analysis (Additional file 1). All the raw sequences were analysed and trimmed using the SeqMan software, Lasergene ver. 7.0 (DNASTAR). Sequences were aligned using the CLUSTAL-W program in MegAlign Lasergene ver. 7.0 (DNASTAR) and exported in FASTA format. Sequence diversity (π), defined as the average number of nucleotide differences per site between two sequences, number of polymorphic sites was determined using the DNAsp ver. 5.0 software. The relationships among the haplotypes of *pvmsa180* were evaluated with the me-dian-joining method using the NETWORK software ver. 4.6.1.2. (Fluxus Technology Ltd., Suffolk, UK). The C-terminus amino acid sequences of PvMSA180 were also separately aligned using Clustal W2 to assess the sequence homology among two human and simian malaria parasites.

### Statistical analysis

Simple scatter regression was applied to construct a standard curve using SigmaPlot (Systat Software Inc., San Jose, CA, USA). Data were analysed using GraphPad Prism (GraphPad, Software, San Diego, CA, USA). The Student’s *t* test was used to assess differences between means. Values of *p* < 0.05 were considered statistically significant. The MeV software (MultiExperiment Viewer, http://www.tm4.org/mev.html) was used to visualize the antibody responses of individual patients to the PvMSA180 domains.

## Results

### Schematic structure of PvMSA180

**Fig. 1:**
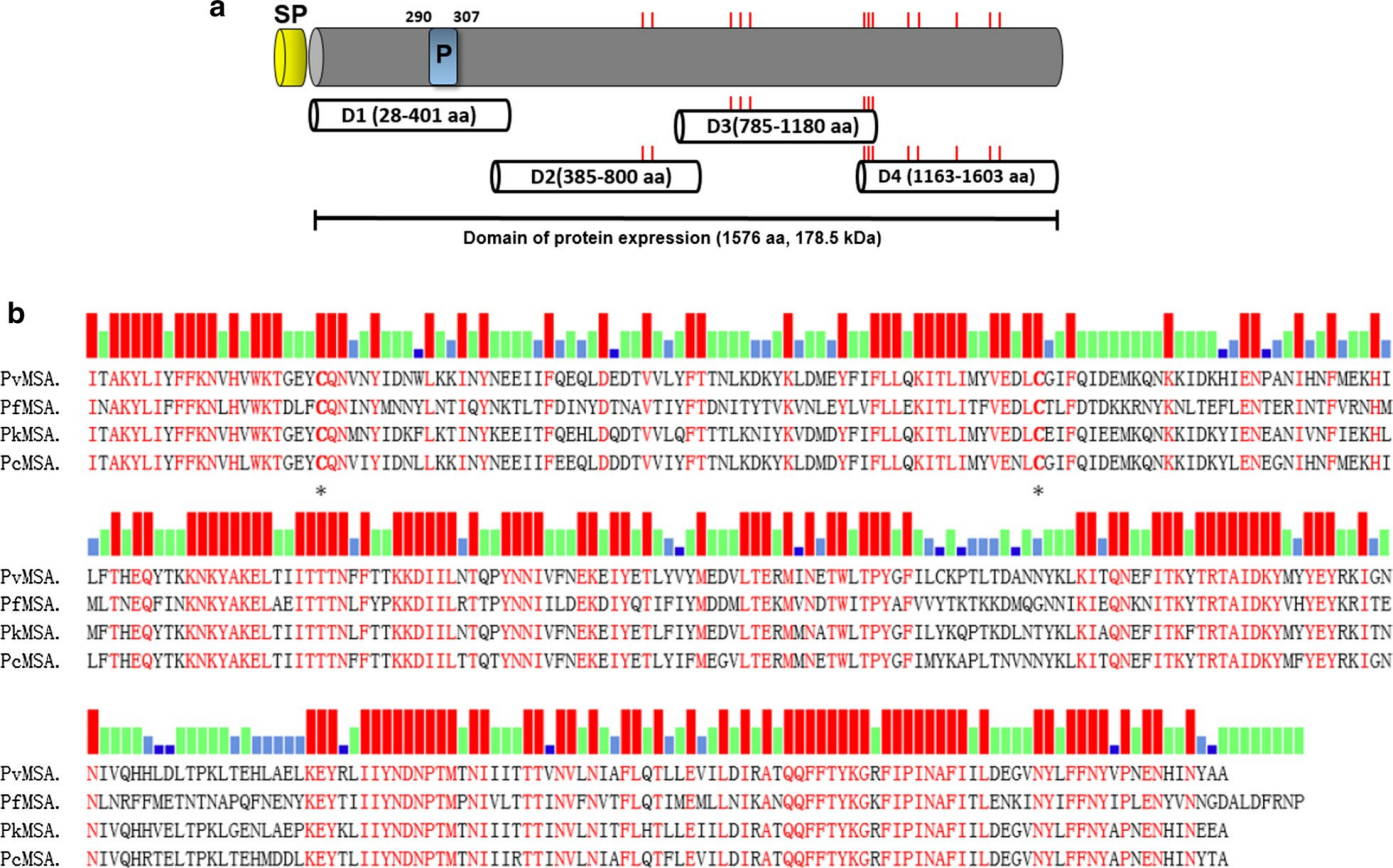
Schematic of recombinant PvMSA180 expression. **a** Schematic of PvMSA180. The D1 (amino acids [aa] 28–401), D2 (aa 385–800), D3 (aa 785–1180), and D4 (aa 1163–1603) domains were expressed using cell-free and bacterial expression systems. *SP* signal peptide, *P* polymorphic region (aa 290–307). **b** Clustal alignment of the C-terminus sequence of MSA180 of *Plasmodium vivax* (Pv), *Plasmodium falciparum* (Pf), *Plasmodium knowlesi* (Pk), and *Plasmodium cynomolgi* (Pc). *Red bars* indicate the conserved aa in the four *Plasmodium* species, *green bars* in three species, *sky*-*blue bars* in two species, and *dark*-*blue bars* in one species. *Asterisks* denote cysteine residues conserved in the four *Plasmodium* species

The protein consists of 1603 amino acids with a predicted molecular mass of 182 kDa, and lacks a transmembrane domain and GPI anchor, as determined using the deduced amino acid sequence of *P*. *vivax* (Sal-1). The cysteine residues are adjacent to the C-terminus domain. Proteins were expressed without a signal peptide. The D1 domain of PvMSA180 (207–307 amino acids) from worldwide isolates contains a polymorphic region (Fig. 1a). In addition, the C-terminus sequence of PvMSA180 has high sequence homology with the human (*P*. *falciparum*) and two simian (*Plasmodium knowlesi* and *Plasmodium cynomolgi*) malaria parasites. The amino acid alignment revealed 70% amino acid identity among human and simian malaria parasites (Fig. 1b).

### Expression of recombinant PvMSA180 proteins

**Fig. 2:**
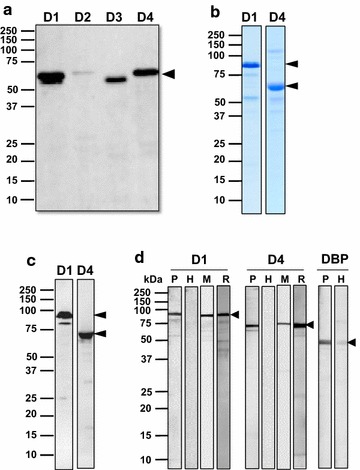
Expression of recombinant PvMSA180. **a** Expression of recombinant domains of PvMSA180 in a wheat germ cell-free expression system. *Arrowheads* indicate the four domains of PvMSA180. *D1* domain 1, *D2* domain 2, *D3* domain 3, and *D4* domain 4. **b** Expression and purification of PvMSA180-D1 (GST-fused) and D4 (His-tagged) in *E*. *coli*. **c** Recombinant PvMSA180-D1 and D4 were probed with anti-GST and anti-His antibodies under reducing conditions. *Arrowheads* indicate the PvMSA180-D1 (~80 kDa) and D4 (~60 kDa) bands. **d** Antibody recognition of PvMSA180-D1 and D4 compare to PvDBP-RII recombinant proteins using pooled vivax-patient sera (*lane P*), malaria naïve healthy sera (*lane H*), mouse immune serum (*lane M*), and anti-GST or anti-His antibody (*lane R*)

The four domains of PvMSA180 were expressed using a wheat germ cell-free system, resulting in a single band of ~50 to 75 kDa; all the domains were predicted to be <50 kDa (Fig. 2a). PvMSA180-D1 was successfully expressed as a 70.3 kDa GST fusion protein, and PvMSA180-D4 was expressed in *E*. *coli* as a 6× His-tagged, 52.7 kDa recombinant protein (Fig. 2b, c). SDS-PAGE and Western blotting showed that recombinant PvMSA180-D1 and PvMSA180-D4 have molecular weights of ~80 and ~60 kDa, respectively (Fig. 2b, c). Purified PvMSA180-D1 and PvMSA180-D4 were used to immunize Balb/c mice with the aim of generating polyclonal antibodies.

### Recognition of recombinant PvMSA180 proteins

To confirm the immunoreactivity of PvMSA180, purified recombinant PvMSA180-D1 and PvMSA180-D4 were incubated with pooled sera of vivax-infected patients. Bands at ~80 and ~60 kDa for PvMSA180-D1 and PvMSA180-D4, respectively, were detected by Western blotting (Fig. 2d). In addition, to confirm antibody production in mice against the D1 and D4 domains of PvMSA180, recombinant proteins were subjected to Western blotting using polyclonal anti-PvMSA180-D1 and PvMSA180-D4 antibodies (Fig. 2d).

### Humoral immune response to PvMSA180

**Fig. 3:**
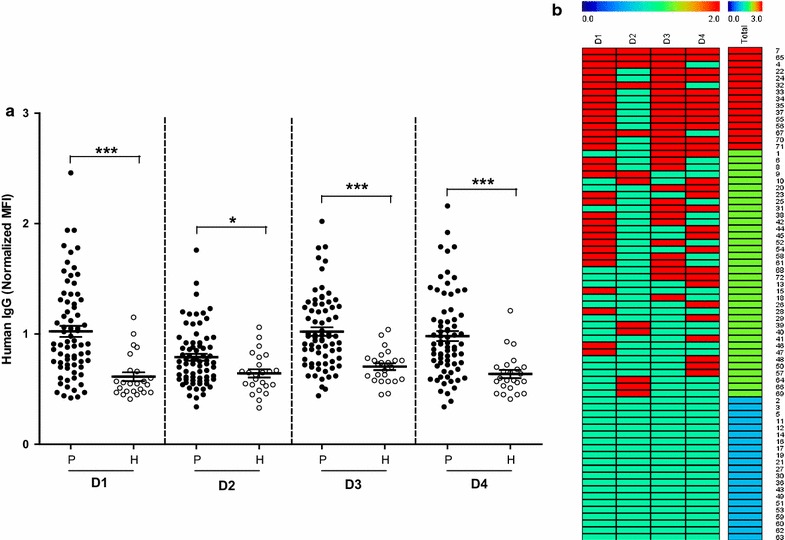
Human IgG response to the four domains of PvMSA180 by protein microarray. **a** Crude recombinant PvMSA180 was probed with sera from vivax-infected patients (P) and naïve healthy individuals (H). The total prevalence of anti-PvMSA180 IgG significantly differed between patients and naïve healthy individuals. *p < 0.01; ***p < 0.0001 by Student’s *t*-test. *Bars* indicate means ± standard deviation. **b** IgG response to the four domains of PvMSA180 in individual patients. *Red* and *green bars* indicate positive and negative reactivity, respectively, in each domain. For total reactivity, *red bar* indicates 3–4 positive domains; *green* indicates 1–2 positive domains, and *blue* indicates the negative domains. The serial numbers of the serum samples are shown on the *vertical line*

The humoral immune response of 72 vivax-infected patients and 24 healthy individuals to the four domains of PvMSA180 was evaluated by protein microarray. Sera from vivax-infected patients showed significantly higher reactivity than that from healthy individuals (Fig. 3a, *p* < 0.0001). The number of healthy individuals and vivax-infected patients showing a serum immune response to PvMSA180 was determined (Table 1). The antibody response to the four domains did not differ markedly. However, antibodies to PvMSA180 showed positive reactivity against PvMSA180, and there was no correlation between the humoral immune response and patient age or the presence of parasitaemia.

**Table 1:** Rate of seropositivity to the four domains of PvMAS180 of vivax-infected patients and naïve controls

Domains	No. of patient samples (*n*)			Normalized MFI^b^	95% CI^d^	No. of healthy samples (*n*)			Normalized MFI^b^	95% CI^d^	*p* value^e^
	Positive	Negative	Total (%)^a^			Positive	Negative	Total (%)^c^			
I	32	40	72 (44.4)	1.02	33.5–55.9	1	23	(22) 95.8	0.61	79.8–99.3	*p* < 0.0001
II	12	60	72 (16.7)	0.79	9.8–26.9	1	23	(22) 95.8	0.64	79.8–99.3	*p* < 0.01
III	29	43	72 (40.3)	1.02	29.7–51.8	1	23	(22) 95.8	0.70	79.8–99.3	*p* < 0.0001
IV	30	42	72 (41.7)	0.98	30.9–53.2	0	24	(24) 100	0.64	86.2–100	*p* < 0.0001

^a^Seropositivity rate: percentage of positive malaria-patient samples

^b^Normalized MFI: mean fluorescence intensities were divided by the cut-off value +2 standard deviations above the mean fluorescence intensity of the malaria-naïve samples

^c^Sero negative rate: percentage of malaria-naïve samples

^d^Confidence intervals

^e^Difference in the total IgG prevalence for each antigen between vivax patients and healthy individuals was calculated by the Student’s *t*-test. A value of *P* < 0.05 was considered statistically significant

### PvMSA180 localized on the merozoite surface

**Fig. 4:**
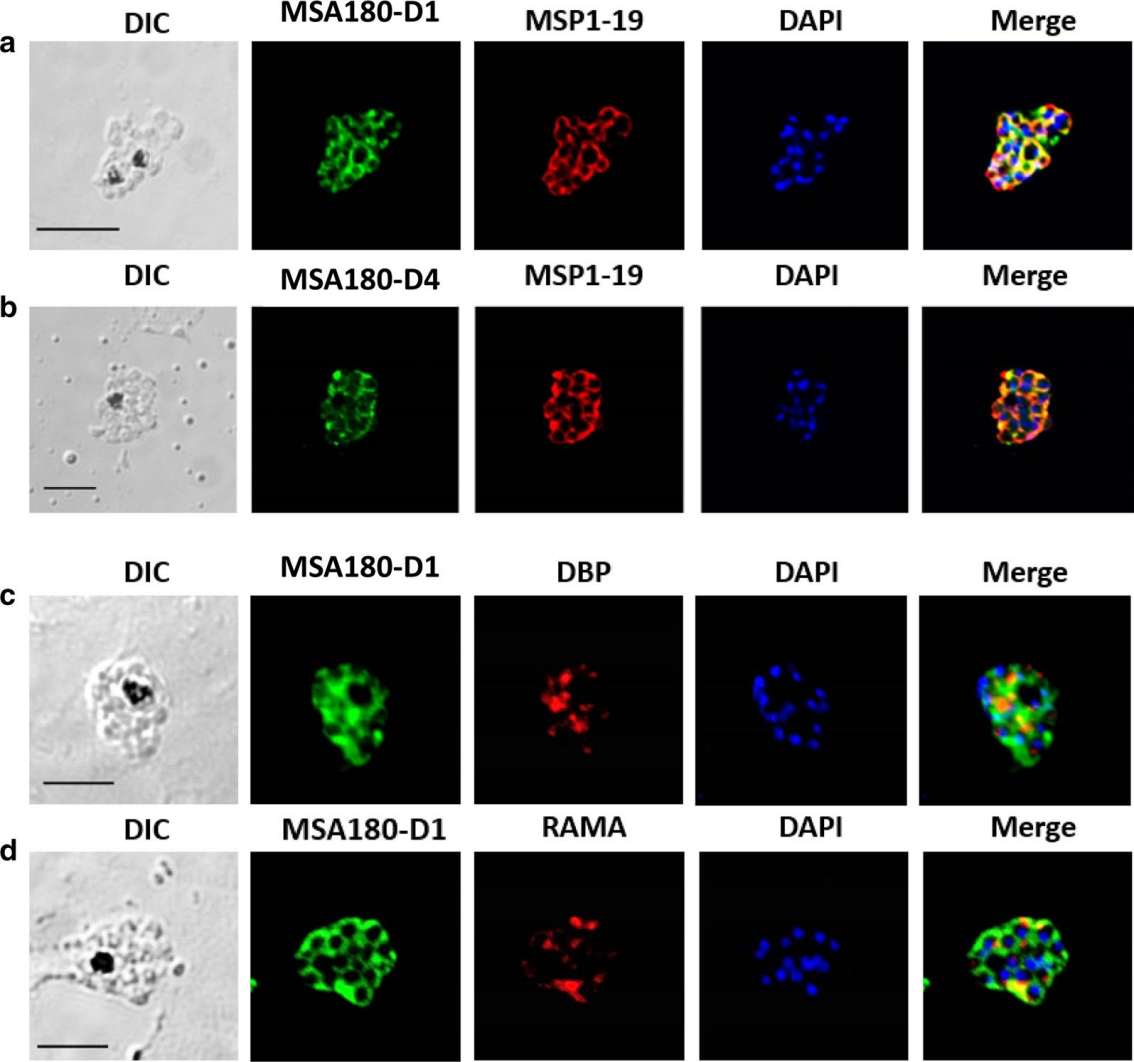
Localization of PvMSA180 in mature schizont-stage parasites. Schizont-stage parasites were dual-labelled with antisera against PvMSP1-19 (merozoite surface marker) (**a** and **b**), PvDBP (microneme marker) (**c**), or PvRAMA (rhoptry marker) (**d**). Nuclei are stained with DAPI in the merged images. *Scale bar* represents 5 µm. *DIC* differential interference contrast

The subcellular localization of PvMSA180 was evaluated by immunofluorescence analysis using an anti-PvMSA180-D1 antibody. Dual-labelled immunofluorescence assays were performed using an anti-PvMSA180-D1 antibody together with an anti-PvMSP1-19 (merozoite surface marker) (Fig. 4a, b), anti-PvDBP (microneme marker) (Fig. 4c), or anti-PvRAMA (rhoptry marker) (Fig. 4d) antibody as controls. The anti-PvMSA180-D1 (N-terminus) and PvMSA180-D4 (C-terminus) antibodies specifically reacted with anti-PvMSP1-19 antibodies in the merozoites of mature schizonts. This suggests that PvMSA180 is localized on the merozoite surface, but not in the rhoptry or microneme.

### Nucleotide diversity and haplotype analysis

**Fig. 5:**
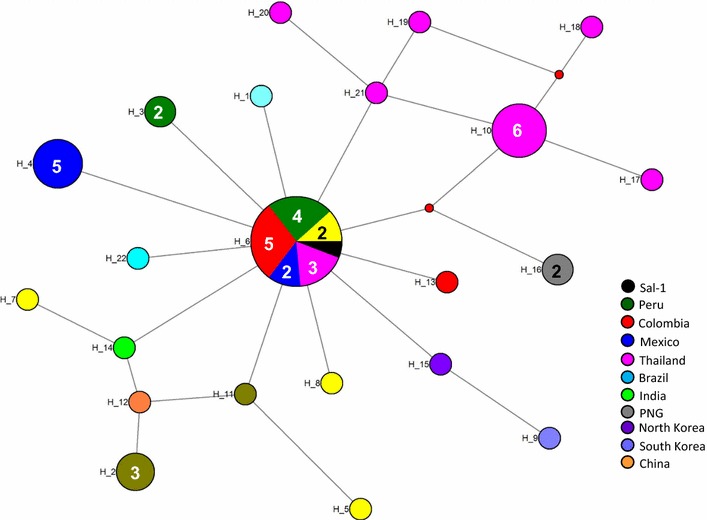
Median-joining networks of *pvmsa180* haplotypes from isolates worldwide. Genealogical haplotype network showing the relationships among 22 *pvmsa180* haplotypes in 51 sequences obtained from *P*. *vivax* isolates from 12 countries. H-number, haplotype number. The *size of the circles* represents the haplotype frequencies and *unnumbered circles* indicate a single haplotype. Geographical haplotypes are indicated by the *colour key*. *Small red nodes* are hypothetical median vectors created by the program to connect sampled haplotypes into a parsimonious network. The distances between nodes are arbitrary

The 51 *pvmsa180* sequences exhibited a low level of diversity (π = 0.00080 ± 0.00012). Alignment of the *pvmsa180* sequences revealed 34 (0.84%) polymorphic and 3985 (99.15%) invariant sites. The polymorphic region (i.e., 879–927 nucleotides) contained the highest nucleotide sequence diversity (π = 0.02196 ± 0.0099). The haplotype network analysis of 51 *pvmsa180* sequences from worldwide isolates identified 22 unique haplotypes with moderate haplotype diversity (Hd = 0.8698). One haplotype (Hap_6) was shared (Fig. 5) by 17 isolates from Colombia (n = 5), Mexico (n = 2), Peru (n = 4), Myanmar (n = 2), Salvador (n = 1), and Thailand (n = 3). None of the other haplotypes were shared (Fig. 5). The haplotypes obtained in this study are listed in Additional file 2.

## Discussion

Genomics, proteomics, and transcriptomics have been used to identify target antigens in *Plasmodium* spp. [10, 21–24]. This has resulted in the identification of proteins expressed by infected red blood cells on the surface or in apical organelles; however, few such *P*. *vivax* proteins have been reported [13]. A small number of promising merozoite antigens has been investigated as vaccine candidates, some of which have shown partial efficacy in clinical trials [13]. MSPs are of interest for the development of vaccines as well as drugs that inhibit blood-stage replication of *P*. *falciparum* [25–27]. In this study, PvMSA180, a novel surface protein of *P*. *vivax*, was characterized.

Most of the cysteine residues are in the C-terminus domain of PvMSA180, which has no transmembrane domain or GPI-anchor. The results of the immunofluorescence assay showed that the PvMSA180 protein is localized on the merozoite surface; thus, it might be categorized as being peripherally associated with the surface of *P*. *vivax* merozoites as it lacks transmembrane domains and a GPI-anchor [13]. PvMSA180 orthologs in other species have a conserved domain at the C-terminus that shows ~70% sequence similarity with simian malaria parasites (*P*. *knowlesi* and *Plasmodium chabaudi*), but a lower level of identity to *Plasmodium falciparum*. Nevertheless, *P*. *vivax* is genetically, phenotypically, and biologically more similar to *P*. *chabaudi* and *P*. *knowlesi* than to *P*. *falciparum* [28–31]. Unlike erythrocyte-binding ligand (*ebl*) and reticulocyte-binding protein (*rbp*), which are crucial for the invasion of erythrocytes by *P*. *vivax*, *P*. *knowlesi*, and *P*. *chabaudi*, the function of *msa180* in other species is unclear.

The amplification and sequence analysis of clinical isolates from diverse geographic regions showed the presence of a short polymorphic domain at the N-terminus region of *pvmsa180*. The polymorphic region of invasion genes is considered under immune selection pressure [32] and plays a role in protective immunity against clinical isolates [32–34]. However, in this study, the polymorphic and conserved domains of PvMSA180 showed similar immunogenicity. Furthermore, 20.8% of the patients (15/72) showed a robust antibody response to more than three domains of PvMSA180, and only 30% did not respond to any of the antigens (Fig. 3b). The development of a malaria vaccine based on merozoite surface antigens has been hindered by their high sequence diversity [35, 36]. In this study, haplotype network analysis resulted in the detection of 22 haplotypes, 1 of which (Hap_6, 17/22) was shared by six countries. However, a greater number of samples are required to confirm this finding, as serum samples from only Myanmar and the ROK were used in this study. These data will facilitate the development of a subunit vaccine based on the domains conserved among strains isolated worldwide. The functional activity of this antibody could not be performed due to the limitation of *P. vivax* culture system. It remains to be determined as further study in the future.

## Conclusions

In this study, a novel *P*. *vivax* surface protein, PvMSA180, was identified. Its immunogenicity in a naturally exposed population suggests that it elicits a humoral immune response, and its localization suggests that it may be involved in merozoite attachment to, and invasion of red blood cells. However, further functional and biological investigations of PvMSA180 protein are required prior to its development as a subunit vaccine. Genetic analysis indicated that the gene is less polymorphic than other well-known candidates and a haplotype common to six countries was identified.

## Additional files

**Additional file 1.** *Pvmsa180* sequence information.

**Additional file 2.** Shared haplotypes.
