# Supplementary material for: Identification of a novel merozoite surface antigen of Plasmodium vivax, PvMSA180
Source: Malar J. 2017 Mar 28;16:133. doi: 10.1186/s12936-017-1760-9 (PMC5369000; doi:10.1186/s12936-017-1760-9)
Supplement: Supplementary file 1 — Additional file 1. Pvmsa180 sequence information. [file 12936_2017_1760_MOESM1_ESM.docx]

**Additional file 1. *Pvmsa180* sequence information**

| **Sample ID** | **Source** | **Country** |
| --- | --- | --- |
| Sal-1 | Reference sequence | Salvador |
| South Korea | Amplified | South Korea |
| Myanmar 088 | Amplified | Myanmar |
| Myanmar 089 | Amplified | Myanmar |
| Myanmar 091 | Amplified | Myanmar |
| Myanmar 092 | Amplified | Myanmar |
| Myanmar 094 | Amplified | Myanmar |
| Brazil I | PlasmoDB | Brazil |
| Brazil 12 | PlasmoDB | Brazil |
| DTS0791 | PlasmoDB | Peru |
| DTS0830 | PlasmoDB | Peru |
| DTS0839 | PlasmoDB | Peru |
| Mexico_63-08 | PlasmoDB | Mexico |
| Mexico_118-A | PlasmoDB | Mexico |
| Mexico_165-A | PlasmoDB | Mexico |
| Mexico_566-A | PlasmoDB | Mexico |
| Mexico_1086-A | PlasmoDB | Mexico |
| Mexico_161-04 | PlasmoDB | Mexico |
| Mexico_203-04 | PlasmoDB | Mexico |
| Peru06 | PlasmoDB | Peru |
| Peru257 | PlasmoDB | Peru |
| Peru1008 | PlasmoDB | Peru |
| North Korean | PlasmoDB | North Korea |
| Thai_VKBT-37 | PlasmoDB | Thailand |
| Thai_VKBT-39 | PlasmoDB | Thailand |
| Thai_VKBT-52 | PlasmoDB | Thailand |
| Thai_VKBT-36 | PlasmoDB | Thailand |
| Thai_VKBT-45 | PlasmoDB | Thailand |
| Thai_VKBT-52 | PlasmoDB | Thailand |
| Thai_VKBT-71 | PlasmoDB | Thailand |
| Thai_VKBT-72 | PlasmoDB | Thailand |
| Thai_VKBT-95 | PlasmoDB | Thailand |
| Thai_VKBT-98 | PlasmoDB | Thailand |
| Thai_VKBT-99 | PlasmoDB | Thailand |
| Thai_VKBT-100 | PlasmoDB | Thailand |
| Thai_VKBT-101 | PlasmoDB | Thailand |
| Thai_VKBT-101 | PlasmoDB | Thailand |
| Thai_VKBT-106 | PlasmoDB | Thailand |
| China_LZCH-4 | PlasmoDB | China |
| China_NB-16 | PlasmoDB | China |
| China_NB-17 | PlasmoDB | China |
| China_LZCH-13 | PlasmoDB | China |
| China_LZCH-20 | PlasmoDB | China |
| Colombia_30101099036 | PlasmoDB | Colombia |
| Colombia_30101099040 | PlasmoDB | Colombia |
| Colombia_30102100437 | PlasmoDB | Colombia |
| Colombia_30102100441-A | PlasmoDB | Colombia |
| Colombia_30102100441-B | PlasmoDB | Colombia |
| Colombia_30102100445 | PlasmoDB | Colombia |
| IndiaVII | PlasmoDB | India |
| PNG58 | PlasmoDB | Papua New Guinea |
| PNG72 | PlasmoDB | Papua New Guinea |
