# Supplementary material for: Identification of a novel merozoite surface antigen of Plasmodium vivax, PvMSA180
Source: Malar J. 2017 Mar 28;16:133. doi: 10.1186/s12936-017-1760-9 (PMC5369000; doi:10.1186/s12936-017-1760-9)
Supplement: Supplementary file 2 — Additional file 2. Shared haplotypes. [file 12936_2017_1760_MOESM2_ESM.docx]

**Additional file 2. Shared haplotypes**

| **Haplotype ID** | **Haplotype** | **Frequency** |
| --- | --- | --- |
| Hap_1 | GGGTCGCACACTACCTCGGCTGCCTATAAGGGTT | 1 |
| Hap_2 | GGGTCACGCACTACCTCGGCGTTTTATAAAGGTT | 3 |
| Hap_3 | GAGTCGCACACTACCTCGGCTGCCTATAAAGGTT | 2 |
| Hap_4 | GGGTCGCACACTATCTCGGCTGCCTATAAAGGTT | 5 |
| Hap_5 | AGATCGCACACTACCTCGGCGTCCTGGAAAGGCT | 1 |
| Hap_6 | GGGTCGCACACTACCTCGGCTGCCTATAAAGGTT | 17 |
| Hap_7 | GGGTCGCGCACTACCTCGGCTGCCTATTGATGTT | 1 |
| Hap_8 | GGGTCGCACACTACCTCGATTGCCTATAAAGGTT | 1 |
| Hap_9 | GGGTCGTACATTACCCCAGCTGCCCATAAAGTTC | 1 |
| Hap_10 | GGGTCGCACGCAACCTCGGCTGCCTATAAAGGTT | 6 |
| Hap_11 | GGGTCGCACACTACCTCGGCGTCCTATAAAGGTT | 1 |
| Hap_12 | GGGTCGCGCACTACCTCGGCGTCCTATAAAGGTT | 1 |
| Hap_13 | GGGTCGCATACTACCTCGGCTGCCTATAAAGGTT | 1 |
| Hap_14 | GGGTCGCGCACTACCTCGGCTGCCTATAAAGGTT | 1 |
| Hap_15 | GGGTCGCACACTACCTCGGCTGCCTATAAAGTTT | 1 |
| Hap_16 | GGGCCGCACACATCCTCGGCTGCCTATAAAGGTT | 2 |
| Hap_17 | GGGTCGCACGCAACTTCGGCTGCCTATAAAGGTT | 1 |
| Hap_18 | GGGTTGCACGCAACCTCGGCGTCCTATAAAGGTT | 1 |
| Hap_19 | GGGTTGCACGCTACCTCGGCTGCCTATAAAGGTT | 1 |
| Hap_20 | GGGTCACACGCTACCTCGGCTGTTTATAAAGGTT | 1 |
| Hap_21 | GGGTCGCACGCTACCTCGGCTGCCTATAAAGGTT | 1 |
| Hap_22 | GGGTCGCACACTACCTTGGCTGCCTATAAAGGTT | 1 |
